# Searching for epistatic interactions in nuclear families using conditional linkage analysis

**DOI:** 10.1186/1471-2156-6-S1-S148

**Published:** 2005-12-30

**Authors:** Svati H Shah, Michael A Schmidt, Hao Mei, William K Scott, Elizabeth R Hauser, Silke Schmidt

**Affiliations:** 1 Center for Human Genetics, Duke University Medical Center, Durham NC, USA

## Abstract

**Background:**

Genomic screens generally employ a single-locus strategy for linkage analysis, but this may have low power in the presence of epistasis. Ordered subsets analysis (OSA) is a method for conditional linkage analysis using continuous covariates.

**Methods:**

We used OSA to evaluate two-locus interactions in the simulated Genetic Analysis Workshop 14 dataset. We used all nuclear families ascertained by Aipotu, Karangar, and Danacaa. Using the single-nucleotide polymorphism map, multipoint affected-sibling-pair (ASP) linkage analysis was performed on all 100 replicates for each chromosome using SIBLINK. OSA was used to examine linkage on each chromosome using LOD scores at each 3-cM location on every other chromosome as covariates. Two methods were used to identify positive results: one searching across the entire covariate chromosome, the other conditioning on location of known disease loci.

**Results:**

Single-locus linkage analysis revealed very high LOD scores for disease loci D1 through D4, with mean LOD scores over 100 replicates ranging from 4.0 to 7.8. Although OSA did not obscure this linkage evidence, it did not detect the simulated interactions between any of the locus pairs. We found inflated type I error rates using the first OSA method, highlighting the need to correct for multiple comparisons. Therefore, using "null chromosome pairs" without simulated disease loci, we calculated a corrected alpha-level.

**Conclusion:**

We were unable to detect two-locus interactions using OSA. This may have been due to lack of incorporation of phenotypic subgroups, or because linkage evidence as summarized by LOD scores performs poorly as an OSA covariate. We found inflated type I error rates, but were able to calculate a corrected alpha-level for future analyses employing this strategy to search for two-locus interactions.

## Background

Genomic screens such as those simulated for the Genetic Analysis Workshop 14 (GAW14) dataset generally employ a "single-locus" search for linkage, in which linkage to a particular marker or set of markers is considered independently from any other locus. Such an approach is quite effective for detecting linkage to loci with strong effects, but may have low power in the presence of epistatic or heterogeneous effects. Two-locus [1,2] and conditional [3] linkage analyses were developed to consider such heterogeneity or epistasis. These methods differ somewhat in their approach, but both attempt to evaluate whether linkage at one locus is influenced by linkage to another. In both cases, there is a need to determine if evidence for linkage under the two-locus or conditional model is stronger than that under the single-locus model. Empirical *p*-values generated by simulation have been used to evaluate results of conditional linkage analysis [3,4].

Ordered subsets analysis (OSA) [5] is a method for conditional linkage analysis using continuous covariates. When evidence for linkage at a second locus is used as the covariate, the method tests whether there is a statistically significant increase in linkage evidence at the first locus conditional on evidence at the second locus. OSA uses a similar approach to that proposed by Cox and colleagues [3], except that the evidence for linkage is maximized over subsets of families rank-ordered by the covariate. We explored the use of OSA as a method for two-locus conditional linkage analysis. We analyzed 300 nuclear families (100 each from Aipotu, Karangar, and Danacaa) for each of the 100 replicates. Having examined the answers prior to beginning our analysis, we focused on six chromosomes harboring disease loci (1–3, 5, 9, 10) and two chromosomes with no disease loci (4, 6).

## Methods

### Dataset

We used all available nuclear families ascertained by three groups: Aipotu, Karangar, and Danacaa. Affection status was determined using the criteria defined by each site; to better approximate a real world post-hoc pooled analysis of three genomic screens, we made no attempt to impose standardized diagnostic criteria. Given there was no genetic heterogeneity in the dataset other than that defined for the three phenotypes, this decision should have introduced some variability in the strength of the main effects to our combined dataset. Introduction of this variability should thereby have attenuated the main effects, allowing weaker epistatic interactions to be detectable. We analyzed each of the 100 replicates. We used the single-nucleotide polymorphism (SNP) linkage map (560 markers spaced at 3 cM on chromosomes 1–3, 5, 9, and 10; and 200 markers spaced at 3 cM on chromosomes 4 and 6). No follow-up markers were ordered.

### Analysis

Multipoint affected sib pair (ASP) linkage analysis was performed using SIBLINK [6]. A grid of ASP LOD scores was generated for each family at 3-cM intervals across each chromosome and used to create a covariate file for use in OSA. OSA initially orders families by family-specific LOD scores at one locus (covariate chromosome). OSA then calculates LOD scores across a second chromosome (analysis chromosome) by summing the family-specific LOD scores at the analysis chromosome, in order of their ranking based on the covariate chromosome. Specifically, for each family *i *a matrix of linkage statistics Z*i*(*d*,γ) is required as input, where *d *represents the disease location parameter and represents the genetic model, and the maximum ordered subset statistic for each family is calculated at a set of values for *d *and γ. OSA begins by ordering *N *number of families by the covariate chromosome family-specific LOD score value x*i*, both in an ascending and a descending order, where Z_(*j*)_(*d*,γ) is the linkage statistic matrix for ordered family *j*. The maximum LOD score is calculated for the *j*^th ^family, as well as the estimates of *d*_(*j*) _and γ_(*j*) _at which the maximum occurs. Then, element-wise addition is used to add the matrix for the next ordered family Z_(j+1)_(*d*,γ) to the matrix for family 1 through *j*. In summary, the *j*^th ^partial sum is created by adding each element of the linkage statistic matrix for each family up to and including ordered family *j*. Addition of each of the N families results in a set of maxima for each partial sum of the linkage statistic (Z^1^(*d*^1^, γ^1^). . . Z^N^(*d*^N^, γ^N^)), ordered by the family-specific covariate value. The final OSA output includes an overall LOD score calculated using all families, a maximum subset LOD score (representing the highest LOD score using subsets of families with the highest covariate chromosome LOD scores), and an estimate of the disease location on the analysis chromosome.

OSA was used to examine linkage on each chromosome with disease loci (analysis chromosome) using LOD scores at each 3-cM location on every other chromosome with disease loci ("covariate" chromosome) as covariates (30 pair-wise evaluations with approximately 100 covariates each). We also performed these same analyses on "null chromosome pairs", using four pairs of chromosomes in which one or both members of the pair did not harbor any disease loci. Families were rank-ordered by decreasing LOD scores on the covariate chromosome in order to highlight potential epistatic interactions. Empirical *p*-values for the increase in the LOD score in the subset of families identified by OSA over baseline LOD scores for the entire dataset were generated, using a minimum of 20 and maximum of 1,000 permutations for pair-wise comparisons of chromosomes with disease loci, and a maximum of 10,000 permutations for the null chromosome pairs. Two methods were used to identify positive OSA results. The first method searched over the entire analysis chromosome for the single most significant OSA LOD score (minimum *p*-value). From the 100 replicates, the number of times that the minimum *p*-value at any position on the analysis chromosome was below 0.01 and 0.05 was calculated. Because we observed a highly inflated type I error rate for the 0.01 and 0.05 significance levels, we calculated a corrected alpha-level for controlling the global type I error rate using OSA results for the null chromosome pairs. The second method conditioned on the exact location of the disease locus on the covariate chromosome and did not suffer from severe type I error inflation. From the 100 replicates, we again calculated the number of times that the OSA empirical *p*-value was below 0.01 and 0.05, and the mean position of the maximum subset-based LOD score on the analysis chromosome.

## Results

To our great surprise, nonparametric multipoint ASP analysis with SIBLINK generated very high LOD scores for D2 on chromosome 3 (297 cM), ranging from 3.2 to 18.1 across the 100 replicates with a mean LOD score of 7.8. Single-locus LOD scores for D3 on chromosome 5 (at 6 cM) were also high, ranging from 1.3 to 9.0, with a mean of 4.1. LOD scores were likewise high for D1 on chromosome 1 (range 1.9–18.6, mean LOD 7.1) and D4 on chromosome 9 (range 0.96–11.4, mean LOD 4.0). As expected, the presence of such strong single-locus results made it difficult to detect significant LOD score increases in family subsets. Lower overall LOD scores were obtained for D5 (chromosome 10, range 0–4.2, mean LOD score 1.0) and D6 (chromosome 2, range 0.1–2.3, mean LOD score 0.8), both of which are disease-modifying genes affecting penetrance of the disease. To simulate a real-world scenario, in which one has no *a priori *knowledge of the disease gene location or of epistatic relationships between loci, we first used the OSA strategy of searching across the entire covariate chromosome for evidence of heterogeneity in the family-specific LOD scores using *p*-value thresholds of 0.01 and 0.05. The proportion of replicates with empirical *p*-values less than 0.01 ranged from 0.19 to 0.52, suggesting a grossly inflated global type I error rate. A Bonferroni correction based on approximately 100 covariate positions analyzed per analysis chromosome (corrected *p*-value: 0.0005) resulted in a more reasonable proportion of replicates with significant results, ranging from 0.02 to 0.18. There appeared to be no relationship between the proportion of replicates with significant results and chromosome pairs reported to harbor epistatically interacting loci (D2 × D3 for P2 and P3 phenotype, D3 × D4 for P2 phenotype, D1 × D4 for P3 phenotype). There was likewise no relationship between the true disease locus position on the covariate chromosome and the position highlighted as significant when using the above *p*-value thresholds. There was little deviation between the position on the analysis chromosome where the OSA LOD score was maximized and the true analysis chromosome disease locus position.

Table 1 displays representative results of the targeted OSA analysis, where we only used ASP LOD scores at the true gene locations on chromosomes with disease loci as OSA covariates. When we used the best LOD scores on chromosomes with disease loci as covariates for chromosomes 4 and 6, neither of which harbor disease loci, evidence for linkage was found slightly more frequently than the significance level, indicating that type I error was slightly inflated even using a targeted approach to conditional linkage analysis. However, more replicates are required to evaluate the distribution of these lower *p*-values. For D1 and D2 (with the highest overall multipoint LOD scores), the OSA LOD score was maximized at (or very close to) the true location of the disease locus, regardless of the covariate chromosome used. For D3, D4, and D5, there was larger variability in the analysis chromosome position, with OSA maximizing the LOD score in general 10–20 cM distal to the actual disease locus. For locus D6, OSA failed to maximize the LOD score at or near the actual disease locus. This is not surprising because D6 is a modifying locus that affects penetrance, and our analyses were restricted to affected individuals only.

Because our targeted analysis is not feasible for real data in which the true disease gene locations are unknown, we focused on calculating a *p*-value threshold (alpha level) to properly correct for testing multiple chromosome pairs with many OSA covariates. To this end, we analyzed four pairs of chromosomes in which one or both members of the pair did *not *harbor any disease loci (Table 2). This resulted in 400 available replicates of "null chromosome pairs" for calculating the corrected *p*-value at which the global type I error rate would be no larger than 5%. These calculations resulted in a corrected alpha level of 0.0006, very similar to a Bonferroni correction despite the fact that the OSA covariates (ASP LOD scores calculated every 3 cM) are presumably correlated with each other.

**Figure 1:**
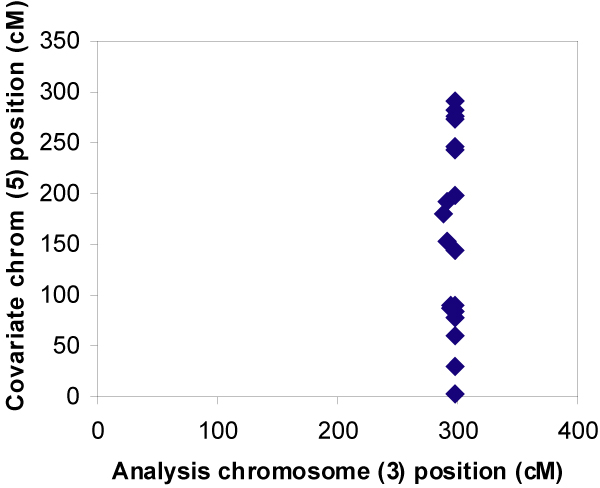
OSA results: chromosome 3 conditional on chromosome 5, replicates with *p*-values < 0.0006.

**Figure 2:**
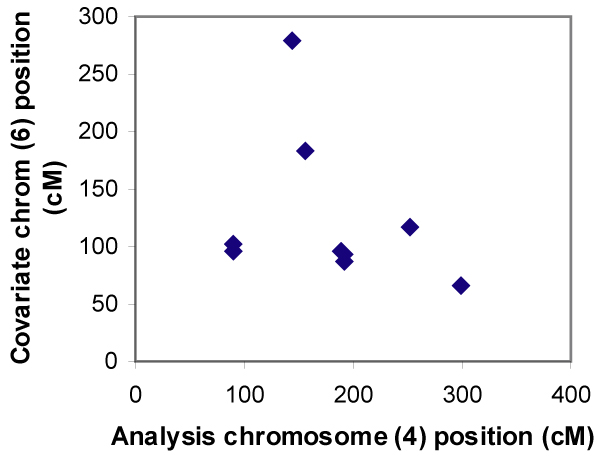
OSA results: chromosome 4 conditional on chromosome 6, replicates with *p*-values < 0.0006.

Conditional linkage analysis did not identify any significant two-locus linkage effects between chromosomes modeled to have epistatic relationships. As shown in Figure 1, significantly increased conditional LOD scores were distributed equally across the covariate position on chromosome 5, implying lack of evidence for an influence of linkage to the D3 region of chromosome 5 on linkage to chromosome 3. A similar pattern existed for results on other disease loci harboring chromosomes (data not shown). The increases in LOD score detected by OSA were not related to linkage at the other disease locus, but rather due to finding random subsets that generated greater LOD scores. When evaluating linkage to chromosome 4 conditional on linkage to chromosome 6 (neither of which contain disease genes), there was random scattering by chromosome and covariate position (Figure 2). A similar random pattern was observed when analyzing linkage to chromosome 6 conditional on chromosome 4.

## Discussion

There are several potential reasons for our failure to detect the epistatic interactions in this dataset. First, family-specific LOD scores as a statistical measure of linkage evidence are likely to be poor surrogates for underlying genotypes with epistatic interactions, and probably have much less variability than measured phenotypic covariates, especially in small nuclear families. Second, we chose to combine the three definitions of Kofendrerd Personality Disorder used by the respective ascertainment sites, but this apparently did not introduce sufficient heterogeneity for OSA to identify genetically (and phenotypically) more homogeneous subsets of families. It may have been more appropriate to incorporate the phenotypic heterogeneity into our analyses and test whether OSA had been able to detect it, or to analyze P1, P2, and P3 separately in order to detect the phenotype-specific epistatic interactions. Third, the strong single-locus effects modeled in the data, the magnitude of which was unknown to us when we embarked on this study, may have diminished our ability to detect the weaker epistatic effects. Table 1 demonstrates that for most interactions, the proportion of families included in the OSA subset was high, suggesting little genetic heterogeneity in the dataset. For the loci with the strongest evidence for linkage, there was little variability in the family-specific LOD scores that could have been used to detect the epistatic interactions. The OSA methodology is best suited for dissecting heterogeneity; in this case, the lack of genetic heterogeneity may be the primary cause of failure of OSA. Furthermore, the delta LOD scores were small because of the strong underlying single-locus effects and compounded by the small size of the dataset, suggesting a lack of power to detect weaker two-locus effects. Given that epistatic interactions existed between loci that themselves had strong evidence for linkage, OSA was not the ideal method to dissect the underlying weaker epistatic effects. However, this issue is likely also generalizable to other methods. Although such strong main effects are unlikely in most complex diseases, the issue is one that investigators should bear in mind. Regardless, our null chromosome analyses were useful in providing data on type I error, and for computing a corrected *p*-value for use in future analyses.

In summary, OSA as a method for conditional linkage analysis did not obscure the readily detected linkage evidence on chromosomes known to harbor disease loci. However, it did not detect any of the simulated epistatic interactions in the GAW14 dataset, nor did it refine the previously well defined locations for the disease genes, primary due to the strong single-locus effects and little genetic heterogeneity modeled in the dataset. OSA remains an important potential tool to evaluate epistatic interactions. Our analyses were useful in allowing us to comment on type I error. In a genome-wide OSA analysis, type I error rates are inflated, indicating that a correction for multiple testing is very important in order to avoid follow-up of false-positive results. By analyzing simulated null chromosomes, we found our corrected alpha level to be 0.0006, which was very similar to a Bonferroni correction for approximately 100 covariate positions evaluated on each analysis chromosome. Thus, if OSA were used on real genome screen data to identify epistatic interactions, this would be a reasonable *p*-value threshold for controlling the global type I error rate.

## Conclusion

Although we failed to identify epistatic interactions using OSA for conditional linkage analysis, potentially due to strong single-locus effects and failure to incorporate KPD endophenotypes, we were able to calculate a corrected alpha level to control the global type I error when conducting such analyses in a genome screen.

## Abbreviations

ASP: Affected sib pair

GAW14: Genetic Analysis Workshop 14

OSA: Ordered subsets analysis

SNP: Single-nucleotide polymorphism

## Authors' contributions

SHS, WKS, ERH, SS contributed to study design, manuscript drafting and revision, and statistical analysis. MAS, HM contributed to statistical analysis. All authors read and approved the final manuscript.

## Figures and Tables

**Table 1:** OSA analysis using peak locations on chromosomes with disease loci as covariates

Chromosome								Proportion of replicates w/empirical *p*-values less than	
							
Analysis (locus)	Covariate (cM)	OSA LOD [Mean (SD)]	Delta LOD^a ^[Mean (SD)]	Proportion of families in OSA subset [Mean (SD)	OSA analysis chromosome position (cM) [Mean (SD)]	Position of Siblink max LOD score (cM) [Mean (SD)]	True position (cM)^b^	0.01	0.05
1 (D1)	**3 (297)**^c^	7.4 (2.7)	0.4 (0.5)	0.93 (0.11)	168.0 (3.9)	167.8 (4.3)	167.9	0	0.03
	**9 (6)**	7.6 (2.7)	0.5 (0.7)	0.92 (0.16)	164.8 (23.0)			0.01	0.04
2 (D6)	**5 (6)**	1.9 (0.6)	1.4 (0.7)	0.28 (0.30)	163.2 (91.2)	143.2 (9.0)	14.8	0.01	0.04
	**9 (6)**	1.9 (0.6)	1.5 (0.7)	0.28 (0.31)	149.5 (86.9)			0	0.01
3 (D2)	**2 (15)**	8.2 (2.5)	0.4 (0.5)	0.92 (0.12)	297.0 (1.7)	296.4 (1.6)	297.7	0.01	0.05
	**5 (6)**	8.3 (2.6)	0.5 (0.6)	0.92 (0.13)	297.1 (1.7)			0.01	0.08
4	3 (297)	1.8 (0.6)	1.3 (0.7)	0.38 (0.29)	165.7 (86.5)		N/A	0.03	0.05
	5 (6)	1.9 (0.6)	1.4 (0.8)	0.26 (0.29)	144.5 (85.6)			0.03	0.07
5 (D3)	**2 (15)**	4.7 (1.8)	0.7 (0.8)	0.79 (0.29)	15.3 (39.9)	7.9 (21.6)	6	0.01	0.04
	**3 (297)**	4.6 (1.8)	0.6 (0.7)	0.80 (0.26)	12.3 (34.9)			0.01	0.07
6	3 (297)	1.8 (0.7)	1.2 (0.7)	0.34 (0.31)	165.7 (93.2)		N/A	0	0.03
	5 (6)	2.0 (0.9)	1.8 (0.9)	0.20 (0.23)	166.1 (92.7)			0	0.03
9 (D4)	**1 (168)**	4.5 (2.0)	0.7 (0.8)	0.79 (0.31)	27.0 (62.7)	13.9 (42)	5.6	0	0.02
	**5 (6)**	4.6 (2.0)	0.8 (0.9)	0.76 (0.31)	38.0 (78.7)			0.01	0.03
10 (D5)	**3 (297)**	1.8 (0.7)	1.1 (0.7)	0.41 (0.34)	81.4 (55.2)	82.4 (53.6)	67	0	0.02
	**5 (6)**	2.0 (0.9)	1.4 (0.8)	0.28 (0.31)	88. 1 (50.9)			0.03	0.07

^a^Change in SIBLINK LOD using OSA.

^b^True disease locus position on analysis chromosome.

^c^ Chromosome pairs in bold reported to have epistatic or genetic modifying interactions.

**Table 2:** OSA analysis of chromosomes without disease loci

Chromosome					Proportion of replicates w/empirical *p*-values less than		
				
Analysis	Covariate	OSA LOD [Mean (SD)]	Delta LOD^a ^[Mean (SD)]	Proportion families in OSA subset [Mean (SD)]	0.0006	0.01	0.05
4	6	4.1 (0.8)	3.9 (0.7)	0.27 (0.15)	0.03	0.40	0.92
6	4	4.2 (0.8)	3.9 (0.7)	0.26 (0.15)	0.05	0.35	0.88
4	5	4.3 (0.7)	4.0 (0.6)	0.27 (0.15)	0.02	0.51	0.97
5	4	6.6 (1.9)	3.5 (1.3)	0.79 (0.16)	0.07	0.36	0.89

^a^Change in SIBLINK LOD using OSA.
